# Cross‐Cultural Validation of the COmprehensive Score for Financial Toxicity (COST) Measure in an Australian Sample

**DOI:** 10.1002/cam4.70779

**Published:** 2025-03-24

**Authors:** Bora Kim, Claudia Rutherford, Lucy Lehane, Judith Fethney, Louise Acret, Tracy King, Patsy Kenny, Richard De Abreu Lourenco, Kate White

**Affiliations:** ^1^ The Daffodil Centre A Joint Venture With Cancer Council, The University of Sydney Camperdown New South Wales Australia; ^2^ Cancer Care Research Unit Sydney Local Health District Camperdown New South Wales Australia; ^3^ Susan Wakil School of Nursing and Midwifery The University of Sydney Camperdown New South Wales Australia; ^4^ Clinical Governance, Quality and Safety Department Chris O'Brien Lifehouse Camperdown New South Wales Australia; ^5^ Institute of Haematology Royal Prince Alfred Hospital Camperdown New South Wales Australia; ^6^ Centre for Health Economics Research and Evaluation (CHERE), University of Technology Sydney Broadway New South Wales Australia

**Keywords:** cancer, cancer treatment, financial distress, financial toxicity, psychometric validation, supportive care

## Abstract

**Introduction:**

The COmprehensive Score for Financial Toxicity (COST) measure developed in the United States measures the financial impact resulting from cancer and its treatment. This paper reports on an Australian cross‐cultural validation of the COST measure.

**Methods:**

Participant eligibility criteria included individuals aged ≥ 18, undergoing or completed primary treatment for cancer. Immediate family members of patients were also eligible for Phase 1. Face and content validity were assessed through concurrent interviews and a brief survey (*Phase 1*). The subsequent revised version of COST was field‐tested using a prospective sample of participants to examine item importance, internal consistency reliability, and construct validity (*Phase 2*).

**Results:**

*Phase 1*: Pretesting included 20 patients and two family members (mean age 61, range: 20–87). 19 or more patients agreed or strongly agreed that the COST items were written in clear language, easy to understand, and relevant to their experiences. Content analysis of interviews led to the inclusion of two family‐related items to improve content coverage (hereby referred to as Australian‐COST), along with two additional stand‐alone items to facilitate clinical actions.

*Phase 2*: One hundred and twenty‐two patients completed a field‐test survey. Each Australian‐COST item was rated as “extremely important” or “important” by most participants (62%–81%). Cronbach's alpha coefficients were > 0.9 for both the COST and the Australian‐COST. Exploratory factor analysis revealed two factors, explaining 64% (COST) and 63% (Australian‐COST) of the variance. Both measures discriminated between low and high household income groups (< $39,999/year, > $125,000/year), demonstrating known‐groups validity (COST: mean 19.51 vs. 28.33, *p* = 0.002, Australian‐COST: mean 23.51 vs. 33.29, *p* = 0.006).

**Conclusions:**

The COST is a valid and reliable measure for Australian cancer patients, and the results largely support the construct equivalence across the United States and Australia. The revised version contains new family‐related items to enhance content coverage, which may be applicable in other countries where the financial impact of cancer on the family is of significant concern for patients.

## 1 Introduction

In 2022, Australia had 162,163 estimated new cancer cases, with a projection of 43% of individuals receiving a cancer diagnosis by the age of 85 [1]. In recent years, there has been increasing awareness of the financial impact cancer has on patients and their families. Cancer‐related financial impact can manifest as reduced spending on meeting basic needs, taking on debt, and selling assets as a way of coping with cancer‐related treatment expense [2, 3]. These financial impacts have been shown to affect psychological health, treatment adherence, and overall health‐related quality of life [4, 5]. Negative financial consequences are not only reported during active treatment [6] but also extend into survivorship [7], with an estimated 45% of patients experiencing financial burdens and related distress after cancer treatment [8].

Researchers have coined the term “financial toxicity” to refer to the objective economic impact of cancer and its treatment, as well as secondary ramifications such as psychological distress [9]. The COmprehensive Score for Financial Toxicity (COST) measure, one of the Functional Assessment of Chronic Illness Therapy (FACIT) suite of measures, was developed to measure financial impacts experienced by cancer patients in the United States (US), enabling standardized assessment in clinical care and research [10]. Version 2 of the COST measure is now available, with an additional stand‐alone summary item assessing the financial hardship of the respondent and their families [11].

The COST measure has been utilized in clinical studies as a screening tool to initiate supportive intervention and as a measure of trial outcomes [12, 13, 14]. The COST has been validated in diverse linguistic groups worldwide, demonstrating its reliability and validity across different cultural contexts and health systems in both solid and hematological cancers [15, 16, 17, 18, 19, 20].

Universal access to publicly funded healthcare is available in Australia, allowing various health care services and medications to be covered under the Medicare Benefits Schedule (MBS) and the Pharmaceutical Benefits Scheme (PBS). Given the differing healthcare systems and funding structures between Australia and the United States, we cannot assume equivalent experiences of cancer‐related financial impact across these countries, warranting cross‐cultural validation of the COST in Australia. Previously, Durber et al. [21] examined the internal consistency reliability, test–retest reliability, and convergent validity of the COST in Australia, but did not examine face and content validity. Assessment of these aspects of validity is crucial for ensuring the construct equivalence of the COST to the experiences of Australian patients with cancer. To fill this gap, a cross‐cultural validation study of the COST was undertaken in an Australian sample, including face and content validity.

## 2 Materials and Methods

### 2.1 Study Design, Participants, and Settings

This prospective, mixed‐methods study was conducted in two sequential phases. Phase 1 assessed the content and face validity of the COST through pretesting methods involving concurrent semistructured interviews and a brief survey. Phase 2 involved field testing with a larger sample of survey participants to examine content validity using item importance, internal consistency reliability, and construct validity. The study design is outlined in Table 1. The Sydney Local Health District Ethics Review Committee (RPAH Zone protocol X16‐0110 and X17‐0274) granted ethics approval for this study. The data were stored in a secure data storage system at the University of Sydney.

**TABLE 1:** Overview of study design.

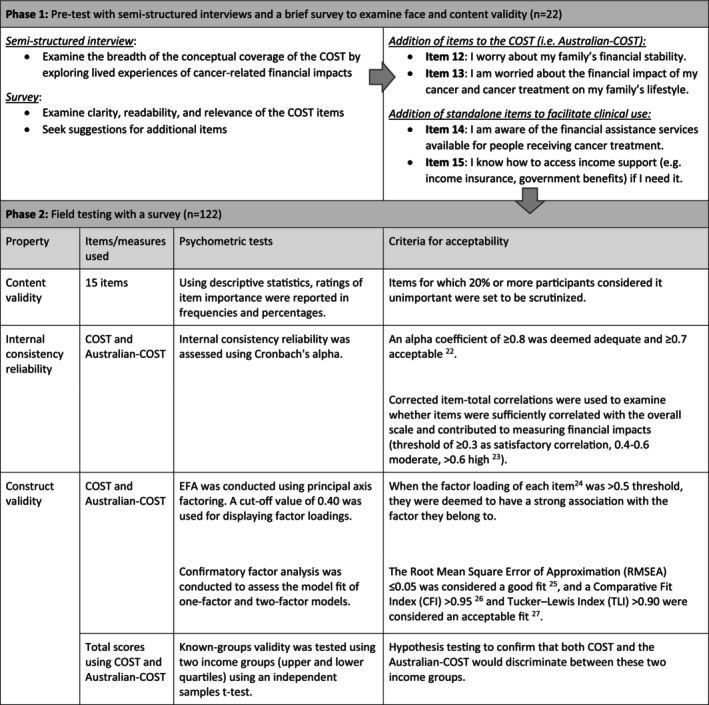

The study was conducted at two cancer centers within tertiary teaching hospitals in Sydney, Australia. Participants were eligible to participate if they were ≥ 18 years, had a cancer diagnosis, and were either currently undergoing or had completed primary treatment. Immediate family members of patients who were ≥ 18 and could share the experience of cancer‐related financial impacts on behalf of the patient were also eligible to participate in Phase 1. Cognitive impairment and non‐English‐speaking status were exclusion criteria for this study.

### 2.2 Measure

For this study, version 1 of the COST, an 11‐item patient‐reported outcome measure designed to assess cancer‐related financial impact, was used. Total scores range from 0 to 44, with lower scores indicating a greater cancer‐related financial impact [28].

### 2.3 Procedures

#### 2.3.1 Recruitment

Study invitation letters for Phase 1 interviews and Phase 2 anonymous surveys were distributed by clinicians and research staff in the clinics and waiting rooms of the treatment suites. For Phase 1, interested participants contacted the research staff directly, and single interviews were conducted in person or via telephone. For Phase 2, those who opted in to complete the anonymous survey returned the completed paper‐based survey using an enclosed prepaid envelope or a sealed box placed in the waiting rooms. Recruitment occurred from September 2016 to February 2017 (Phase 1), and March 2018 to February 2019 (Phase 2).

#### 2.3.2 Phase 1: Pretest Using Interviews and a Brief Survey

To assess whether the COST adequately covered all significant financial impacts resulting from cancer in Australia, semistructured interviews were conducted. Participants were asked to share their lived experiences with the following topics: (1) financial impacts of cancer and its treatment; (2) types of out‐of‐pocket costs; (3) changes to household income; and (4) if financial challenges were experienced, how they were managed and what could have helped them manage these challenges.

Participants were then asked to self‐complete the COST measure and rate its clarity, readability, and relevance to their experience (Data S1), using a Likert scale ranging from “strongly agree” (5) to “strongly disagree” (1), along with their verbal rationale for the given scores. To assess content coverage, participants were also asked to comment on any items that should be added to reflect their experiences related to the financial impact of cancer.

#### 2.3.3 Phase 2: Field Test Using a Survey

Based on findings from Phase 1, a modified version of COST with two additional family‐related items to improve content coverage (totaling 13 items, hereby referred to as Australian‐COST) was used for field testing, along with two stand‐alone items added measuring knowledge of support services to facilitate clinical actions (Data S1). The survey contained Australian‐COST to allow psychometric testing, followed by a section asking participants to rate the importance of each item using a Likert scale with responses ranging from “extremely important” (4) to “not important” (1).

### 2.4 Data Analysis

#### 2.4.1 Phase 1. Pretesting: Content and Face Validity

The qualitative data describing participants' lived experiences with the financial impacts of cancer were analyzed using content analysis [29]. One researcher inductively developed textual units by coding and categorizing interview data and examined their relevance to each COST item. Another researcher verified the identified mapping of the data to the COST item by cross‐checking the codes and the data. Any discrepancies were resolved through discussions. The major themes not covered by the COST measure were brought to the team for further discussion to examine the need for adding new items. The brief survey responses relating to item clarity, readability, relevance, and content coverage were summarized and reported descriptively.

#### 2.4.2 Phase 2. Field Testing: Internal Consistency Reliability, Content and Construct Validity

Descriptive statistics were used to describe the study participants, total COST and Australian‐COST scores, and the perceived item importance (as part of content validity), which were reported as percentages, frequencies, means, and standard deviations (SD) as appropriate. The total financial impact scores for both COST and Australian‐COST were calculated by summing each item, multiplying by 11, and dividing by the total number of valid responses, conforming to the current COST scoring manual [11]. Items 2, 3, 4, 5, 8, 9, 10, 12, and 13 were reverse‐coded; thus, a lower total score indicated a greater financial impact [10, 11].

Internal consistency reliability and construct validity were examined for both the COST and Australian‐COST. Internal consistency reliability was examined using Cronbach's alpha coefficients. Construct validity was examined through factor analyses. Exploratory factor analysis (EFA) conformed to the method reported in the original development of the measure [10], specifically principal axis factoring. A cutoff value of 0.40 was used for displaying factor loadings. Confirmatory factor analysis (CFA) was conducted to assess the model fit of the factor structures of the COST and Australian‐COST. Known‐groups validity was tested using independent samples t‐tests to compare two groups based on their household income before tax [16, 21]; those below $39,999 per year and those at $125,000 or above per year, guided by the household income quartiles in New South Wales in 2016 [30].

For exploratory purposes, sociodemographic factors associated with the total Australian‐COST scores were explored using Analysis of variance (ANOVA). Predictor variables included participant age, sex, cancer type, marital status, working status, and health insurance type. All statistical analyses were conducted using IBM SPSS version 28, except for the CFA, which was analyzed using AMOS version 29. All items had 8% or fewer missing cases (median: 2, range: 0–10), where each participant's mean of all nonmissing items was substituted.

## 3 Results

### 3.1 Phase 1. Pretesting: Content and Face Validity

Twenty patients and two family members participated in the interviews, with a mean participant age of 61, and the majority were female (*n* = 14) (Table 2).

**TABLE 2:** Interview participant characteristics.

Characteristic	Total *N* = 22 (%)
Patient or Carer/family member	
Patient	20 (90.9)
Carer/family member	2 (9.1)
Daughter	1 (4.5)
Wife	1 (4.5)
Sex	
Male	8 (36.4)
Female	14 (63.6)
Marital status^a^	
Married	14 (63.6)
De facto	1 (4.5)
Single	3 (13.6)
Separated/divorced	4 (18.2)
Current work status^a^	
Paid work	7 (31.8)
Self‐employed	4 (18.2)
Retired	7 (31.8)
Look after home/family, studying	2 (9.1)
Unemployed	2 (9.1)
Has your current work status changed since diagnosis?^a^	
Yes	10 (45.5)
No	12 (54.5)
Primary cancer type^a^	
Multiple myeloma	10 (45.5)
Gastrointestinal	5 (22.7)
Lymphoma	3 (13.6)
Leukemia	2 (9.1)
Melanoma	1 (4.5)
Breast cancer	1 (4.5)
Time since primary cancer diagnosis^a^	
Less than 1 year	3 (13.6)
1–4 yours	5 (22.7)
5–10 years	3 (13.6)
Over 10 years	10 (45.5)
Not specified	1 (4.5)
Treatment modalities^a^, ^b^	
Surgery	8 (36.4)
Radiotherapy	6 (27.3)
Transplant	12 (54.5)
Chemotherapy	20 (90.9)
Completion of cancer treatment^a^	
Completed	7 (31.8)
Ongoing	13 (59.1)
Not specified	2 (9.1)
Insurance or benefits^a^, ^b^	
Private Health Insurance—with extras	9 (40.9)
Private Health Insurance—without extras	2 (9.1)
Healthcare concession card	8 (36.4)
Department of Veterans Affairs Gold or White card	0 (0.0)
None	5 (22.7)
Annual household income before taxes^a^	
$10,000–$59,999 per year	11 (50.0)
($190–$1149 per week)	
$60,000–$149,999 per year	4 (18.2)
($1150–$2879 per week)	
$150,000–$200,000 or more per year	4 (18.2)
($2880–$3840 or more per week)	
Do not know	3 (13.6)

^a^ Family participant provided demographic and clinical information on behalf of their family member with cancer.

^b^ Multiple selections were possible.

In the brief survey, most participants either agreed or strongly agreed that the COST measure was written in clear language (*n* = 19), not difficult to understand (*n* = 21), and relevant to their experiences (*n* = 20) (Data S1). In open verbal feedback, most expressed that the COST measure captured relevant experiences relating to the financial impact of cancer and its treatment. Eleven participants suggested additional items to the measure, including family‐related financial concerns. A summary of verbal feedback on the COST is presented in Data S1.

Participants lived experiences pertaining to the financial impact of cancer showed that all 11 items of COST were relevant to the experiences of our Australian participants. However, a prominent and consistent concern described by participants across the interviews was the financial impacts of cancer and its treatment on their families, which were not adequately reflected in version 1 of COST. For example, there was a greater concern for parents with cancer about the financial compromises their children had to make. Others also feared burdening their spouse and their spouses' financial prospects in the case of their death.

To cover the family‐related financial impacts in the COST, we reinstated one of the candidate items that was not included in the original development of the measure [10], as well as one new item derived from our qualitative analysis (Items 12 and 13, Data S1).

Several participants also emphasized the importance of having practical support, such as information provision, when one experienced financial difficulty. This was particularly true for those whose mental energy was depleted, as one described, “you don't have energy to do anything let alone say,Let's see if we can ring these people and find out [what financial assistance might be available]. ” (Female patient with Multiple Myeloma). Not knowing about eligible financial support early in their cancer journey was also an issue, for example, “We had nothing to do with Centrelink [prior to cancer diagnosis], so it was quite hard to go through the process, not knowing exactly how we qualified, what we qualified for; we didn't even know Mum could get Carer's Leave, so there was quite a lot [financial assistance] that came to us a lot later” (Daughter of a patient with lymphoma).

To address these gaps, two additional stand‐alone items were added, measuring knowledge of support services to help identify patients who may have information gap. The intention was to use them in conjunction with the total financial impact score to facilitate the provision of information (Items 14 and 15, Data S1).

### 3.2 Phase 2. Field Testing: Internal Consistency Reliability, Content and Construct Validity

One hundred and twenty‐two respondents completed the survey; the mean age was 61 (SD 15.13), 50% males, and multiple myeloma was the predominant cancer type (42.6%) (Table 3). The mean total score was 24.1 (SD 10.77, range 0–44) using the COST and 28.5 (SD 12.79, range 0–52) using the Australian‐COST. Greater financial impact was reported by participants aged < 75 years (compared to aged ≥ 75), females, those with solid tumors (compared to hematological cancer), those without any health insurance or with a healthcare concession card, divorced/separated/or widowed individuals, and those who were not in paid work. Comparisons of the total financial impact scores (based on the Australian‐COST) between different demographic groups are presented in Data S1.

**TABLE 3:** Survey participant characteristics.

Variable	Total *N* 122 (%)
Sex	
Female	56 (45.9)
Male	61 (50.0)
Not specified	5 (4.1)
Age	
< 50	19 (15.6)
51–64	31 (25.4)
65–75	31 (25.4)
> 75	12 (9.8)
Not specified	29 (23.8)
Cancer type	
Multiple myeloma	52 (42.6)
Breast	11 (9.0)
Lymphoma	10 (8.2)
Genitourinary	7 (5.7)
Lung	7 (5.7)
Mesothelioma	4 (3.3)
Upper gastrointestinal	3 (2.5)
Leukemia	3 (2.5)
Colorectal	1 (0.8)
Gynecological	1 (0.8)
Others	2 (1.6)
Cancer type not specified	21 (17.2)
Primary treatment type^**a**^	
Chemotherapy	98 (80.3)
Transplant	38 (31.1)
Radiotherapy	31 (25.4)
Surgery	33 (27.0)
Currently receiving treatment^**b**^	
Yes	77 (63.1)
No	26 (21.3)
Not answered	19 (15.6)
Length since cancer diagnosis	
≤ 1 year	26 (21.3)
> 1 year	85 (69.7)
Not specified	11 (9.0)
Marital status	
Married/de facto	81 (66.4)
Divorced/separated/widowed	20 (16.4)
Never married	16 (13.1)
Not specified	5 (4.1)
Work status	
Working full or part‐time, self‐employed	43 (35.2)
Not working^c^	12 (9.8)
Retired	54 (44.3)
Look after home/family studying	6 (4.9)
Other	7 (5.7)
Work status has changed since cancer diagnosis	
Yes	54 (44.3)
No	60 (49.2)
Not specified	8 (6.6)
Health insurance	
Private health insurance—with extras	64 (52.5)
Private health insurance—without extras	6 (4.9)
Healthcare concession card	2 (1.6)
Department of Veterans Affairs Gold or White card	1 (0.8)
None of the above	24 (19.7)
Not specified	7 (5.7)
Annual household income before taxes	
Zero income	3 (2.5)
$10,000–$59,999 per year	37 (30.3)
($190–$1149 per week)	
$60,000–$149,999 per year	40 (32.8)
($1150–$2879 per week)	
$150,000–$200,000 or more per year	15 (12.3)
$2880–$3840 or more per week	
Do not know	27 (22.1)

^a^ Multiple selections were possible.

^b^ Includes ongoing maintenance treatment.

^c^ Unable to work due to disability or sickness, unemployed, unpaid work.

### 3.3 Content Validity

Most participants (62%–81%) rated each of the 15 items as either “extremely important” or “important” (Data S1). Notably, the additional items (Items 12–15) were rated as “extremely important” by 43% to 47% of participants, placing them in the higher range compared to other items, which ranged from 26% to 58% of responses.

### 3.4 Internal Consistency Reliability

Cronbach's alpha coefficients for the COST and Australian‐COST were 0.90 and 0.92, respectively, demonstrating strong internal consistency reliability in both measures. The removal of additional Items 12 and 13 from the Australian‐COST reduced Cronbach's alpha by a small degree to 0.91 (Table 4).

**TABLE 4:** Reliability indices for the COST (11‐item) and the Australian‐COST (13‐item).

Item	Questions	COST Cronbach's α = 0.901		Australian‐COST Cronbach's α = 0.920	
		Corrected item‐total correlation	Cronbach's α if item deleted	Corrected item‐total correlation	Cronbach's α if item deleted
1	I know that I have enough money in savings, retirement, or assets to cover the costs of my treatment	0.52	0.90	0.52	0.92
2	My out‐of‐pocket medical expenses are more than I thought they would be	0.54	0.90	0.54	0.92
3	I worry about the financial problems I will have in the future as a result of my illness or treatment	0.70	0.89	0.72	0.91
4	I feel I have no choice about the amount of money I spend on care	0.50	0.90	0.51	0.92
5	I am frustrated that I cannot work or contribute as much as I usually do	0.63	0.89	0.64	0.92
6	I am satisfied with my current financial situation	0.76	0.89	0.75	0.91
7	I am able to meet my monthly expenses	0.65	0.89	0.63	0.92
8	I feel financially stressed	0.80	0.88	0.81	0.91
9	I am concerned about keeping my job and income	0.61	0.89	0.63	0.92
10	My cancer or treatment has reduced my satisfaction with my present financial situation	0.71	0.89	0.72	0.91
11	I feel in control of my financial situation	0.64	0.89	0.62	0.92
12	I worry about my family's financial stability			0.72	0.91
13	I am worried about the financial impact of my cancer and cancer treatment on my family's lifestyle			0.76	0.91

The corrected item‐total correlations for the COST and the Australian‐COST ranged between 0.50–0.76 and 0.51–0.81, respectively, indicating a strong correlation with the total score, thus demonstrating high internal consistency reliability (Table 4). Specifically, Items 12 and 13 showed high corrected correlations with the total Australian‐COST score (0.72 and 0.76), suggesting that these items contributed to measuring financial impact.

### 3.5 Construct Validity

#### 3.5.1 Factor Analysis

Exploratory factor analysis supported a two‐factor structure for both COST and Australian‐COST (Data S1). The cumulative variances explained by the first two factors were 64.0% (COST) and 63.0% (Australian‐COST). All items demonstrated factor loadings > 0.5.

CFA demonstrated that the model fit between the single‐factor model of the COST from the original US validation sample [10] and that of the single‐factor model from the current Australian sample was similar in most indices, with the exception of a lower RMSEA in the Australian sample, indicating a better model fit (Table 5). Both measures (COST and Australian‐COST) exhibited acceptable indices (as defined in Table 1) in both 1‐ and 2‐factor models in the Australian sample.

**TABLE 5:** Comparison of the factor structure of COST (11 item) and Australian‐COST (13 item) using confirmatory factor analysis.

COST versions		# of factors	Chi‐square/df	CFI	TLI	RMSEA	GFI	% variance
COST^a^		1	2.4	0.94	0.93	0.08	—	98%
COST^b^, ^c^		1	1.01	1.00	1.0	0.01	0.95	51%
Australian‐COST^b^, ^c^		1	1.22	0.99	0.98	0.04	0.93	52%
COST^b^, ^c^		2	1.15	0.99	0.99	0.04	0.94	64%
Australian‐COST^b^, ^c^		2	1.16	0.99	0.99	0.04	0.93	63%

^a^ Confirmatory factor analysis findings reported in the original development study by De Souza et al. [10] using a US sample.

^b^ Allowing for correlations between error terms specified in modification indices.

^c^ Confirmatory factor analysis was conducted using an Australian sample.

#### 3.5.2 Known‐Groups Testing

The COST and Australian‐COST discriminated between two income groups (below $39,999 per year vs. $125,000 and above per year); COST (Mean 19.51 vs. 28.33, difference = 8.81; 95% CI 3.36, 14.27, *p* = 0.002), and the Australian‐COST (Mean 23.51 vs. 33.29, difference = 9.78; 95% CI 2.97, 16.59, *p* = 0.006), with the lower income groups having lower scores (greater financial impact).

## 4 Discussion

The 11‐item COST version 1, developed in the United States by De Souza et al. [10], was found to be a valid and reliable measure of financial impact that was overall relevant and representative of the experiences of Australian cancer patients. Our findings largely supported construct equivalence across the two countries. However, the pretesting of COST in Australian patients led to the addition of four items to enhance its relevance and facilitate supportive care actions, making it suitable for the Australian context. The suggested use of the Australian‐COST is summarized in Data S1.

The content and face validity of the COST were demonstrated in Phase 1. The items were highly rated for clarity, readability, and content relevance. Semistructured interviews demonstrated content coverage and relevance. In Phase 2, internal consistency reliability was high for both COST and Australian‐COST, consistent with findings from the original development [10] and subsequent validation study [28], as well as other cross‐cultural validations [17, 21, 31], including one done in Australia [21]. Corrected item‐total correlations indicated that all 13 Australian‐COST items contributed to measuring financial impact. EFA found a two‐factor model, supporting this model as a better fit in both COST and Australian‐COST compared to the one‐factor model identified in the original development [10]. Several other studies also found the two‐factor model more suitable, such as in Brazil [32], Korea [17], and China [16]. The Australian‐COST discriminated between patients in low‐ and high‐income brackets as hypothesized and consistent with other studies [16, 21].

Our pretesting led to the addition of two new items to the COST covering family‐related financial concerns, which now contribute to the total scoring of the Australian‐COST. Notably, these two items were among those rated “extremely important” by our survey participants. Another study that developed a financial impact measure specifically for radiation oncology patients also identified the financial concerns for families as one important dimension of cancer‐related financial impact, thus forming part of their measure [33]. After our study completion, an updated version of the COST (Version 2) was released with a new standalone summary item measuring the financial hardship experienced by the respondents and their families [11], further reinforcing the rationale for including family‐related items. The nature of cancer‐related financial impact on families can range from lifestyle adjustments [34] to more serious household material hardship, such as food, energy, and housing insecurity [35], as well as family members forgoing medical care for their own health conditions [36]. The Australian‐COST is intended for the Australian context, but its use may also be relevant to cancer care in other countries with a similar cultural mix, health care, and social support systems; this warrants further research.

Our pretest findings also led to the addition of two stand‐alone items that assess respondents' knowledge of financial assistance services and income support to facilitate clinical action. There is emerging evidence suggesting that routine assessment of financial impact and referrals to financial counseling and navigation programs were feasible [14, 37] and effective in improving health‐related quality of life and survival outcomes [38]. In Australia, most oncologists address treatment expenses with fewer than half of their patients, despite recognizing their importance [39]. Completion of Items 14 and 15 could facilitate clinical actions by better linking patients with existing financial assistance services and income support that are available in Australia when information gaps may exist.

## 5 Limitations

The majority of the participants in our study had multiple myeloma; thus, our participants may not be representative of the general cancer cohort. However, previous studies have validated the COST across various cancer types [40], indicating evidence of its reliability and validity in a broad range of cancer types. Future validation can be conducted on more diverse and underrepresented samples, such as people living in nonmetropolitan areas and migrant populations, given the challenges of additional travel‐related expenses and barriers to accessing support services. Continued discussion is warranted to examine the need for tumor or treatment‐specific measures [33] while weighing the benefit of having a generic measure for international comparisons across tumor and treatment types.

## 6 Conclusion

This study showed that the COST was a valid and reliable measure of financial impact for Australian cancer patients and demonstrated construct equivalence between the United States and Australia. The Australian version contains two new family‐related items to enhance its content coverage to reflect the experiences of Australian cancer patients. This revised version may also be applicable in other countries where family‐related financial impact is a significant concern for patients, warranting more research for further validation.

## Author Contributions

**Bora Kim:** formal analysis (lead), methodology (equal), writing – original draft (lead). **Claudia Rutherford:** formal analysis (lead), methodology (equal), supervision (equal), writing – review and editing (equal). **Lucy Lehane:** conceptualization (equal), data curation (lead), formal analysis (lead), methodology (equal), project administration (lead), writing – review and editing (equal). **Judith Fethney:** formal analysis (lead), methodology (equal), supervision (equal), writing – review and editing (equal). **Louise Acret:** data curation (equal), formal analysis (equal), project administration (equal), writing – review and editing (equal). **Tracy King:** conceptualization (equal), data curation (equal), methodology (equal), writing – review and editing (equal). **Patsy Kenny:** conceptualization (equal), formal analysis (equal), methodology (equal), supervision (equal), writing – review and editing (equal). **Richard De Abreu Lourenco:** conceptualization (equal), formal analysis (equal), methodology (equal), supervision (equal), writing – review and editing (equal). **Kate White:** conceptualization (lead), formal analysis (equal), methodology (equal), supervision (lead), writing – review and editing (equal).

## Ethics Statement

The Sydney Local Health District Ethics Review Committee (RPAH Zone protocol X16‐0110 and X17‐0274) granted ethics approval for this study.

## Consent

All interview participants in Phase 1 of this study provided informed written consent prior to their participation. For Phase 2, the completion and return of the anonymous survey were considered as providing implied consent, as detailed in the patient information sheet enclosed with the copy of the survey.

## Conflicts of Interest

The authors declare no conflicts of interest.

## Supporting information


Data S1.

## Data Availability

The data supporting this study cannot be publicly shared due to ethical or privacy reasons, but it may be shared upon reasonable request to the corresponding author if appropriate.

## References

[1] Cancer Australia , “Cancer Statistics,” n.d. Australian Government., https://www.canceraustralia.gov.au/impacted‐cancer/what‐cancer/cancer‐australia‐statistics#:~:text=It%20is%20estimated%20that%20around,by%20the%20age%20of%2085.

[2] S. Lee , R. G. Olvera , K. Shiu‐Yee , et al., “Short‐Term and Long‐Term Financial Toxicity From Breast Cancer Treatment: A Qualitative Study,” Supportive Care in Cancer 32 (2024): 24. 10.1007/s00520-023-08199-z 38095729

[3] S. L. Schröder , N. Schumann , A. Fink , and M. Richter , “Coping Mechanisms for Financial Toxicity: A Qualitative Study of Cancer Patients' Experiences in Germany,” Supportive Care in Cancer 28 (2020): 1131–1139. 31201545 10.1007/s00520-019-04915-w

[4] C. Benedict , S. Fisher , L. Schapira , et al., “Greater Financial Toxicity Relates to Greater Distress and Worse Quality of Life Among Breast and Gynecologic Cancer Survivors,” Psycho‐Oncology 31 (2022): 9–20. 34224603 10.1002/pon.5763 PMC9809212

[5] N. Bhoo‐Pathy , C.‐W. Ng , G. C.‐C. Lim , et al., “Financial Toxicity After Cancer in a Setting With Universal Health Coverage: A Call for Urgent Action,” Journal of Oncology Practice 15 (2019): e537–e546. 31112479 10.1200/JOP.18.00619

[6] P. M. Carrera , H. M. Kantarjian , and V. S. Blinder , “The Financial Burden and Distress of Patients With Cancer: Understanding and Stepping‐Up Action on the Financial Toxicity of Cancer Treatment,” Cancer Journal for Clinicians 68 (2018): 153–165. 10.3322/caac.21443 PMC6652174 29338071

[7] F. Mols , B. Tomalin , A. Pearce , B. Kaambwa , and B. Koczwara , “Financial Toxicity and Employment Status in Cancer Survivors. A Systematic Literature Review,” Supportive Care in Cancer 28 (2020): 5693–5708. 32865673 10.1007/s00520-020-05719-z PMC7686183

[8] H. Jiang , J. Lyu , W. Mou , et al., “Prevalence and Risk Factors of Self‐Reported Financial Toxicity in Cancer Survivors: A Systematic Review and Meta‐Analyses,” Journal of Psychosocial Oncology 41 (2023): 457–474. 36370039 10.1080/07347332.2022.2142877

[9] L. G. Gordon , K. M. D. Merollini , and A. Lowe , “Financial Toxicity—What It Is and How to Measure It,” Cancer Forum 41 (2017): 30–35.

[10] J. A. De Souza , B. J. Yap , and F. J. Hlubocky , “The Development of a Financial Toxicity Patient‐Reported Outcome in Cancer: The COST Measure,” Cancer 120 (2014): 3245–3253. 24954526 10.1002/cncr.28814

[11] FACIT Group , “COST: A FACIT Measure of Financial Toxicity”, https://www.facit.org/measures/facit‐cost.

[12] M. Doherty , J. Heintz , A. Leader , D. Wittenburg , and J. Jacoby , “Guaranteed Income and Financial Treatment Trial (GIFT Trial or GIFTT): A 12‐Month, Randomized Controlled Trial to Compare the Effectiveness of Monthly Unconditional Cash Transfers to Treatment as Usual in Reducing Financial Toxicity in People With Cancer Who Have Low Incomes,” Frontiers in Psychology 14 (2023): 1320743. 38152561 10.3389/fpsyg.2023.1320743 PMC10751360

[13] C. Alacevich , A. M. Abi Nehme , J.‐H. Lee , et al., “A Point‐of‐Care Pilot Randomized Intervention to Connect Patients With Cancer‐Induced Financial Toxicity to Telehealth Financial Counseling,” Cancer Causes & Control 35 (2024): 393–403. 37794203 10.1007/s10552-023-01794-9 PMC10872295

[14] D. A. Parikh , G. M. Rodriguez , M. Ragavan , et al., “Lay Healthcare Worker Financial Toxicity Intervention: A Pilot Financial Toxicity Screening and Referral Program,” Supportive Care in Cancer 32 (2024): 161. 38366165 10.1007/s00520-024-08357-x

[15] C. I. Ripamonti , F. Chiesi , and P. Di Pede , “The Validation of the Italian Version of the COmprehensive Score for Financial Toxicity (COST),” Supportive Care in Cancer 28 (2020): 4477–4485. 31925533 10.1007/s00520-019-05286-y

[16] H.‐H. Yu , Z.‐F. Yu , and H. Li , “The Comprehensive Score for Financial Toxicity in China: Validation and Responsiveness,” Journal of Pain and Symptom Management 61 (2021): 1297–1304. 33412268 10.1016/j.jpainsymman.2020.12.021

[17] S. Shim , D. Kang , and N. Kim , “Validation of Korean Version of the COmprehensive Score for Financial Toxicity (COST) Among Breast Cancer Survivors,” Cancer Research and Treatment 54 (2022): 834–841. 34645130 10.4143/crt.2021.784 PMC9296937

[18] N. Mejri , H. Rachdi , A. Mnif , et al., “Translation and Validation of the Comprehensive Score of Financial Toxicity for Cancer Patients Into Arabic,” Journal of Nursing Measurement 30, no. 4 (2022): 673–682. 34518422 10.1891/JNM-D-20-00140

[19] E. C. Fradelos , P. M. Prapa , and K. Tsaras , The Validation of the COmprehensive Score for Financial Toxicity (COST) Scale in Greek Language (Springer, 2022), 191–197. 10.1007/978-3-031-31986-0_18 37581793

[20] A. Joshi , D. Kalra , N. Menon , et al., “Translation and Validation of COST—FACIT (Version 2) Questionnaire Into Hindi and Marathi to Assess Financial Toxicity in Indian Cancer Patients,” South Asian J Cancer 11, no. 2 (2022): 97–104. 36466977 10.1055/s-0041-1741074 PMC9718603

[21] K. Durber , G. K. Halkett , M. McMullen , and A. K. Nowak , “Measuring Financial Toxicity in Australian Cancer Patients—Validation of the COmprehensive Score for Financial Toxicity (FACT COST) Measuring Financial Toxicity in Australian Cancer Patients,” Asia‐Pacific Journal of Clinical Oncology 17, no. 4 (2021): 377–387, 10.1111/ajco.13508. 33567158

[22] EORTC Quality of Life Group , “Guidelines for Developing Questionnaire Modules”, https://www.eortc.org/app/uploads/sites/2/2018/02/guidelines_for_developing_questionnaire‐_final.pdf.

[23] P. M. Fayers and D. Machin , Quality of Life: The Assessment, Analysis and Interpretation of Patient‐Reported Outcomes (John Wiley & Sons, 2013).

[24] J. Hair , B. Babin , R. Anderson , and W. Black , Multivariate Data Analysis (Cengage, 2018).

[25] L. R. Fabrigar , D. T. Wegener , R. C. MacCallum , and E. J. Strahan , “Evaluating the Use of Exploratory Factor Analysis in Psychological Research,” Psychological Methods 4, no. 3 (1999): 272–299, 10.1037/1082-989X.4.3.272.

[26] L. Hu and P. M. Bentler , “Cutoff Criteria for Fit Indexes in Covariance Structure Analysis: Conventional Criteria Versus New Alternatives,” Structural Equation Modeling: A Multidisciplinary Journal 6 (1999): 1–55.

[27] P. M. Bentler and D. G. Bonett , “Significance Tests and Goodness of Fit in the Analysis of Covariance Structures,” Psychological Bulletin 88, no. 3 (1980): 588–606.

[28] J. A. De Souza , B. J. Yap , and K. Wroblewski , “Measuring Financial Toxicity as a Clinically Relevant Patient‐Reported Outcome: The Validation of the COmprehensive Score for Financial Toxicity (COST),” Cancer 123 (2017): 476–484. 27716900 10.1002/cncr.30369 PMC5298039

[29] K. Krippendorff , Content Analysis: An Introduction to Its Methodology (Sage Publications, 2018).

[30] City of Sydney , “City of Sydney: Census Data Notes”.

[31] V. V. Sakti , M. Danaee , C.‐H. Yip , et al., “Financial Toxicity Following Cancer in a Middle‐Income Country With a Pluralistic Health System: Validation of the COST Questionnaire,” Cancer Care Research Online 3 (2023): e044.

[32] L. de Alcantara Nogueira , F. J. Koller , L. Marcondes , et al., “Validation of the Comprehensive Score for Financial Toxicity for Brazilian Culture,” Ecancermedicalscience 14 (2020): 1158, 10.3332/ecancer.2020.1158. 33574903 PMC7864683

[33] M. A. Dar , R. Chauhan , K. Murti , V. Trivedi , and S. Dhingra , “Development and Validation of Subjective Financial Distress Questionnaire (SFDQ): A Patient Reported Outcome Measure for Assessment of Financial Toxicity Among Radiation Oncology Patients,” Frontiers in Oncology 11 (2022): 819313. 35186720 10.3389/fonc.2021.819313 PMC8847677

[34] G. Sadigh , J. Switchenko , K. E. Weaver , et al., “Correlates of Financial Toxicity in Adult Cancer Patients and Their Informal Caregivers,” Supportive Care in Cancer 30 (2022): 217–225. 34255179 10.1007/s00520-021-06424-1 PMC8639637

[35] E. M. Evans , J. Lin , J. Sanchez‐Alvarez , A. K. Agrawal , and L. E. Winestone , “Disparities in Household Material Hardship, Financial Toxicity, and Income Loss in Pediatric Cancer,” Pediatric Blood & Cancer 70 (2023): e30496. 10.1002/pbc.30496 37394628

[36] B. Kazzi , F. Chino , B. Kazzi , et al., “Shared Burden: The Association Between Cancer Diagnosis, Financial Toxicity, and Healthcare Cost‐Related Coping Mechanisms by Family Members of Non‐Elderly Patients in the USA,” Supportive Care in Cancer 30 (2022): 8905–8917. 35877007 10.1007/s00520-022-07234-9

[37] J. Edward , K. D. Northrip , M. K. Rayens , et al., “Financial‐Legal Navigation Reduces Financial Toxicity of Pediatric, Adolescent, and Young Adult Cancers,” JNCI Cancer Spectrum 8, no. 3 (2024): pkae025, 10.1093/jncics/pkae025. 38552323 PMC11087728

[38] T. G. Knight , M. Aguiar , and M. Robinson , “Financial Toxicity Intervention Improves Outcomes in Patients With Hematologic Malignancy,” Journal of Clinical Oncology Practice 18, no. 9 (2022): e1494–e1504. 35709421 10.1200/OP.22.00056

[39] A. Agarwal , D. J. Karikios , M. R. Stockler , and R. L. Morton , “Discussion of Costs and Financial Burden in Clinical Practice: A Survey of Medical Oncologists in Australia,” PLoS One 17 (2022): e0273620. 36269711 10.1371/journal.pone.0273620 PMC9586404

[40] Z. Zhu , W. Xing , H. Wen , et al., “Psychometric Properties of Self‐Reported Financial Toxicity Measures in Cancer Survivors: A Systematic Review,” BMJ Open 12 (2022): e057215. 10.1136/bmjopen-2021-057215 PMC9234804 35750459

