# Supplementary material for: Cross‐Cultural Validation of the COmprehensive Score for Financial Toxicity (COST) Measure in an Australian Sample
Source: Cancer Med. 2025 Mar 24;14(6):e70779. doi: 10.1002/cam4.70779 (PMC11931400; doi:10.1002/cam4.70779)
Supplement: Supplementary file 1 — Data S1. [file CAM4-14-e70779-s001.docx]

| **Supplementary A**. Interview participants’ ratings of the clarity, readability and relevance of COST items | | |
| --- | --- | --- |
| Questions | Responses | N (%) |
| The language used in the questions was clear | Strongly agree | 12 (54.5) |
|  | Agree | 7 (31.8) |
|  | Neither agree nor disagree | 1 (4.5) |
|  | Disagree | 2 (9.1) |
|  | Strongly disagree | 0 (0) |
| The questions were not difficult to understand | Strongly agree | 12 (54.5) |
|  | Agree | 9 (40.9) |
|  | Neither agree nor disagree | 1 (4.5) |
|  | Disagree | 0 (0) |
|  | Strongly disagree | 0 (0) |
| The questions were relevant to my experience | Strongly agree | 9 (40.9) |
|  | Agree | 11 (50) |
|  | Neither agree nor disagree | 2 (9.1) |
|  | Disagree | 0 (0) |
|  | Strongly disagree | 0 (0) |

**Supplementary B**. Overview of the Australian-COST and two standalone items

| Item number | Description | Suggested use |
| --- | --- | --- |
| 1-11 | Original 11-item COmprehensive Score for financial Toxicity (COST) | To generate summative financial impact scores for multi-country studies for comparison. |
| 12  (additional item) | ‘I worry about my family’s financial stability’ | Items added to make original COST suitable for use in the Australian context. For Australian-specific studies, summative scores can be generated using items 1-13 (13-item Australian COST). |
| 13  (additional item) | ‘I am worried about the financial impact of my cancer and cancer treatment on my family’s lifestyle’ |  |
| 14  (additional item) | ‘I am aware of the financial assistance services available for people receiving cancer treatment’ | Stand-alone items are intended to be used in conjunction with the summative financial impact scores to facilitate the provision of information on available services or financial support when information gaps exist. |
| 15  (additional item) | ‘I know how to access income support (e.g. income insurance, government benefits) if I need it’ |  |

| **Supplementary C**: Summary of verbal feedback on the COST measure | |
| --- | --- |
| Questions (n of verbal responses)^a^ | Summary of responses |
| Relevance of each item to their experience  (n=20) | Overall, all items were relevant to the experiences of most study participants. Five participants identified some irrelevant items. Specifically, item 2 was not applicable to some participants who had already completed their treatment and no longer had out-of-pocket medical expenses. Some participants had already retired and therefore did not have a job to keep, making the wording 'job' in item 9 not entirely relevant. |
| Suggestions for additional items to the COST measure (n=19) | Eleven participants suggested additional items to the original COST. These included items about coping behaviours, challenges related to debts, stress arising from unforeseen expenses, and concerns about the financial impact of cancer on their family. Some also thought the tool should ask whether the patient knew about financial assistance. Eight participants felt no additional items were required. |
| a. The number of patients providing verbal responses was less than 22 due to time constraints during the interview. | |

| **Supplementary D**: Total Australian-COST item scores by participant characteristics | | | | |
| --- | --- | --- | --- | --- |
| **Variable** | N^a^ | Mean^b^ (SE) | Mean difference | P-value ^d^ |
| **Sex** |  |  |  |  |
| Female | 56 | 24.51 (1.67) | -7.37 (-11.96, -2.78) | .002 |
| Male (ref) | 61 | 31.87 (1.60) |  |  |
| **Age** |  |  |  |  |
| Less than 50 | 19 | 24.91 (3.01) | -13.79 (-23.40, -4.18) | .005 |
| 51-64 | 31 | 27.79 (2.36) | -10.91 (-19.77, -2.05) | .02 |
| 65-75 | 31 | 26.80 (2.36) | -11.91 (-20.76, -3.05) | .009 |
| Over 75 (ref) | 12 | 38.70 (3.79) |  |  |
| **Cancer type** |  |  |  |  |
| Hematology | 65 | 30.91 (1.60) | 5.86 (.45, 11.28) | .034 |
| Oncology (ref) | 34 | 25.04 (2.21) |  |  |
| **Employment status** |  |  |  |  |
| Look after home/family, studying | 6 | 20.17 (4.98) | -7.73 (-18.26, 2.79) | .15 |
| Not working^c^ | 12 | 18.54 (3.52) | -9.36 (-17.25, -1.48) | .02 |
| Retired | 54 | 32.14 (1.66) | 4.24 (-0.70, 9.18) | .09 |
| Working, self-employed (ref) | 43 | 27.90 (1.86) |  |  |
| **Marital status** |  |  |  |  |
| Divorced, separated, widowed | 20 | 21.17 (2.86) | -8.82 (-15.15, -2.50) | .007 |
| Never married | 16 | 27.84 (3.20) | -2.16 (-9.08, 4.77) | .54 |
| Married/de facto (ref) | 81 | 29.99 (1.42) |  |  |
| **Type of health insurance** |  |  |  |  |
| Private health insurance-without extras | 6 | 26.83 (4.99) | -5.30 (-15.65, 5.05) | .31 |
| Healthcare concession card | 20 | 23.63 (2.74) | -8.50 (-14.71, -2.29) | .008 |
| Department of Veterans Affairs card or White card | 1 | 25.00 (12.23) | -7.13 (-31.56, 17.29) | .56 |
| None of these | 24 | 22.52 (2.50) | -9.62 (-15.42, -3.82) | .001 |
| Private health insurance with extras (ref) | 64 | 32.13 (1.53) |  |  |
| a. N includes valid cases only  b. Generated by using the Australian-COST, with a lower score indicating the worse financial impact.  c. Unable to work due to disability/sickness, unemployed, unpaid work  d. The mean difference is significant at the .05 level | | | | |

**Supplementary E**. Item importance ratings for the 15 items

| **Supplementary F**. Comparison of the COST and the Australian-COST using exploratory factor analysis (principal axis factoring analysis, direct oblimin rotation^a^) | | | | |
| --- | --- | --- | --- | --- |
|  | **COST** | | **Australian COST** | |
| Item | Factor 1  Loading | Factor 2  Loading | Factor 1  Loading | Factor 2  Loading |
| **^b^ 13** I am worried about the financial impact of my cancer and cancer treatment on my family’s lifestyle. |  |  | .79 |  |
| **^b^ 12** I worry about my family’s financial stability. |  |  | .75 |  |
| **^b^ 3** I worry about the financial problems I will have in the future as a result of my illness or treatment. | .68 |  | .71 |  |
| **^b^ 4** I feel I have no choice about the amount of money I spend on care. | .75 |  | .69 |  |
| **^b^ 10** My cancer or treatment has reduced my satisfaction with my present financial situation. | .64 |  | .68 |  |
| **^b^ 8** I feel financially stressed. | .66 |  | .68 |  |
| **^b^ 9** I am concerned about keeping my job and income. | .60 |  | .66 |  |
| **^b^ 2** My out-of-pocket medical expenses are more than I thought they would be. | .69 |  | .65 |  |
| **^b^ 5** I am frustrated that I cannot work or contribute as much as I usually do. | .52 |  | .55 |  |
| **7** I am able to meet my monthly expenses. |  | -.80 |  | .82 |
| **1** I know that I have enough money in savings, retirement, or assets to cover the costs of my treatment. |  | -.79 |  | .78 |
| **11** I feel in control of my financial situation. |  | -.69 |  | .69 |
| **6** I am satisfied with my current financial situation. |  | -.69 |  | .66 |
|  | 51.3 % of the variance explained by 1 factor  64.0 % of the variance explained by 2 factors  Cronbach’s α = 0.90 | | 52.0% of the variance explained by 1 factor  63.0 % of the variance explained by 2 factors  Cronbach’s α = 0.92 | |
| a. The Kaiser-Meyer-Oklin value was 0.898 for the COST and 0.890 for the Australian-COST, and the Bartlett test of sphericity demonstrated statistical significance (both < 0.001), suggesting that both measures were suitable for factor analysis.  b. Reverse-scored items | | | | |
